# Genome Sequence of *Candida tropicalis* no. 121, Used for RNA Production

**DOI:** 10.1128/genomeA.00316-14

**Published:** 2014-05-15

**Authors:** Bingbing Li, Ting Guo, Yong Chen, Jingjing Xie, Huanqing Niu, Dong Liu, Jian Cheng, Xiaochun Chen, Jinglan Wu, Wei Zhuang, Chenjie Zhu, Hanjie Ying

**Affiliations:** a State Key Laboratory of Materials-Oriented Chemical Engineering, College of Life Science and Pharmaceutical Engineering, Nanjing Tech University, Nanjing, China; b National Engineering Technique Research Center for Biotechnology, Nanjing Tech University, Nanjing, China; c Guangzhou Sugarcane Industry Research Institute, Guangzhou, China; d Nanjing Biotogether Co. Ltd., Nanjing, China

## Abstract

We report here the complete genome sequence of *Candida tropicalis* no. 121. *C. tropicalis* no. 121 is a high-RNA-producing strain obtained by mutagenesis in our laboratory. The complete genome sequence was determined using the Illumina HiSeq 2000 and contains 6,415 genes. The genome size of *C. tropicalis* no. 121 is >15.3 Mb.

## GENOME ANNOUNCEMENT

*Candida tropicalis* belongs to the genus *Candida* and is a diploid yeast that does not sexually reproduce (1). *C. tropicalis* is one of the more common *Candida* spp. causing human diseases in tropical countries. It is of medical, academic, and industrial interest. In the industrial field, *C. tropicalis* has been used for the production of long-chain dicarboxylic acids (2) and xylitol (3) and induced peroxisomal enzyme expression involved in the utilization of *n*-alkanes (4). In our study, *C. tropicalis* no. 121 was a high-yield strain used for RNA production in our laboratory and Nanjing BioTogether Co. Ltd. Under optimal conditions, the maximum RNA and dry cell weight (DCW) concentrations were >2.5 and 15 g liter^-1^ (5, 6), higher than those for *Kluyveromyces marxianus* (7). At the present, the genome sequence of *C. tropicalis* MYA-3404 is available at the National Center for Biotechnology Information (NCBI) (8). It contains 6,441 genes and has a genome size of 14.63 Mb.

Whole-genome sequencing of *C. tropicalis* no. 121 was performed using the high-throughput sequencing Illumina HiSeq 2000 platform (Beijing Genomics Institute [BGI], Shenzhen, China) by generating short-insert DNA libraries (500 bp, 6 kb, and 10 kb). The read length was 90 bp for these three libraries, resulting in 1,072 Mb to 477 Mb raw data. After the removal of adaptors, low-quality reads, poly-N sequences, error paired-end reads, and duplications, the clean data were assembled using SOAP*denovo* version 1.05 and generated 150 contigs of >1,000 bp, with a total length of 14,979,840 bp. The total size of the resulting assembly is 15,326,821 bp, distributed on 150 scaffolds. The G+C content of the complete genome is 33.11%. The finished results were analyzed and annotated using GeneWise, SNAP, Genemarkers, RNAmmer, and tRNA-SE. The gene function annotation was predicted by using the Kyoto Encyclopedia of Genes and Genomes (KEGG), Clusters of Orthologous Groups (COG), Swiss-Prot, and Gene Ontology (GO).

The complete genome sequence of *C. tropicalis* no. 121 contains 6,415 coding sequences (CDSs), with an average length of 1,462.01 bp, and the total length of the CDSs is 9,378,801 bp. Among the 6,415 genes with an average length of 1,486.53 bp, the numbers of exons and introns are 6,929 and 514, respectively. There is no rRNA predicted by *de novo* prediction. Two hundred thirteen tRNAs, 49 small nuclear RNAs (snRNAs), and 2 micro-RNAs (miRNAs) were annotated and make up 0.1151%, 0.039%, and 0.0012% of the genome, respectively. In addition, there are 547,234-bp repeat sequences that were found in the genome based on the methods of Repbase, ProMask, De novo, and Tandem Repeats Finder (TRF). Furthermore, 217,480-bp transposons were involved in the whole genome.

### Nucleotide sequence accession number.

This whole-genome shotgun project for *C. tropicalis* no. 121 has been deposited at DDBJ/EMBL/GenBank under the accession no. JGYC00000000.
